# Avoiding short circuits from zinc metal dendrites in anode by backside-plating configuration

**DOI:** 10.1038/ncomms11801

**Published:** 2016-06-06

**Authors:** Shougo Higashi, Seok Woo Lee, Jang Soo Lee, Kensuke Takechi, Yi Cui

**Affiliations:** 1 Department of Materials Science and Engineering, Stanford University, 476 Lomita Mall, McCullough Building 343, Stanford, California 94305, USA; 2 Smart Design of Materials and Process Research-Domain, Toyota Central R&D Laboratories, Inc., 41-1 Nagakute, Aichi 480-1192, Japan; 3 School of Electrical and Electronic Engineering, Nanyang Technological University, 50 Nanyang Avenue, Singapore 639798, Singapore; 4 SLAC National Accelerator Laboratory, Stanford Institute for Materials and Energy Sciences, 2575 Sand Hill Road, Menlo Park, California 94025, USA

## Abstract

Portable power sources and grid-scale storage both require batteries combining high energy density and low cost. Zinc metal battery systems are attractive due to the low cost of zinc and its high charge-storage capacity. However, under repeated plating and stripping, zinc metal anodes undergo a well-known problem, zinc dendrite formation, causing internal shorting. Here we show a backside-plating configuration that enables long-term cycling of zinc metal batteries without shorting. We demonstrate 800 stable cycles of nickel–zinc batteries with good power rate (20 mA cm^−2^, 20 C rate for our anodes). Such a backside-plating method can be applied to not only zinc metal systems but also other metal-based electrodes suffering from internal short circuits.

## Abstract (web summary)

Zinc-based aqueous battery chemistries allow for attractive cost and energy densities, but are susceptible to zinc dendrite formation during plating and internal shorting. Here, the authors show that by plating only on the side away from the counter-electrode, internal shorts can be circumvented.

## Graphical abstract

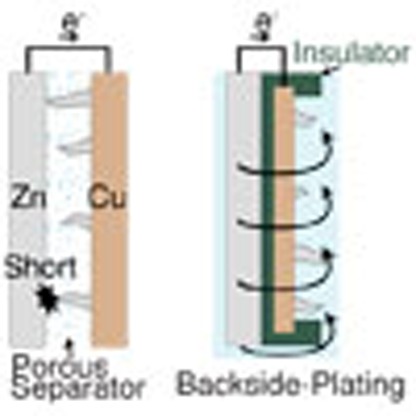

Rechargeable batteries in various forms are critical as power sources for portable electronics, electrical vehicles and grid-scale storages^1^,^2^,^3^,^4^,^5^. For very large-scale deployment, battery materials ideally need to be abundant, accessible and environmentally friendly. Zn satisfies the above criteria and is also recyclable^6^. It has an attractive electrochemical potential as an anode in aqueous battery systems (1.83 V versus Li, −1.245 V versus standard hydrogen electrode in alkaline electrolyte) and possesses high capacity (5,854 Ah l^−1^ and 820 Ah kg^−1^). Zn-based batteries offer the highest energy density of all aqueous battery systems at low cost. In contrast to other high-energy-density metals such as lithium (Li) and sodium, Zn is chemically stable in air and non-flammable. As the invention of the Volta pile (Zn-H_2_O system), which generated continuous current for the first time in early nineteenth century, many battery systems using Zn metal as anode were proposed, such as Zn-NiOOH (Ni-Zn), Zn–air, Zn-MnO_2_ and Zn-Ag_2_O, and some were successfully commercialized as primary battery systems^7^. However, despite past efforts dedicated to making rechargeable Zn-based batteries, stable electrode operation in practical cells has still been a challenge. Unlike the anodes of Li-ion batteries where ions are intercalated and de-intercalated from a graphite host structure, Zn metal anode is a hostless electrode in which the metal dissolves into electrolyte at discharge and plated back at charge, accompanying redistribution of the Zn metal. On charging, dendritic Zn is formed easily and can cause internal short circuits in an unpredictable manner^8^,^9^,^10^,^11^,^12^. Although an internal short circuit in Zn-based batteries does not result in the same hazardous situation as in Li-ion batteries, uncontrolled energy release remains a safety concern. In addition, cycle life can be shortened due to internal shorts^13^. Recently, Zn-based battery systems have been re-visited with a material design of porous Zn metal sponge, demonstrating improved cyclability in Ag-Zn cells (50 cycles with 3–5 mA cm^−2^ of current densities)^14^. A high-energy, high-power cathode for Ni-Zn rechargeable battery has also been demonstrated with nanoscale material design of the Ni hydroxide cathode^15^,^16^. Zn–air batteries with a metal-free bifunctional catalyst show a stable cycling at a current density of 2 mA cm^−2^ (ref. 5). Despite the above progress, the dendrite issue under a range of current densities still remains a critical concern.

Here we tackle the issue of dendrite-induced shorting by developing a concept of backside metal plating. We use half-cells to illustrate our concept as shown in Fig. 1. Figure 1a shows a conventional Zn metal foil electrode, which serves as both reference and counter electrodes, directly facing the Cu working electrode. During Zn plating onto Cu foil in the conventional frontside plating configuration, Zn dendrites can grow towards the Zn reference electrode, causing battery shorting. In our concept demonstrated here (Fig. 1b), the backside plating of Zn is realized by coating an insulating layer on the edges and the ‘front' surface of Cu foil facing the Zn metal counter electrode. Thus, during Zn plating, Zn ions in the aqueous electrolyte travel over the edge and are deposited on the open back surface of Cu foil. Therefore, even if Zn dendrites form, they grow away from the counter electrode and do not short a battery. Looking into the ionic pathway in back plating configuration, Zn-related ions need to go around the insulating layer, to access the back surface of Cu foil. This configuration might seem to be a disadvantage, as it would cause a decrease of the rate capability. However, we note that the high ionic conductivity of 6 M aqueous KOH (0.6 S cm^−1^)^17^, a common electrolyte for conventional Zn-based batteries, can afford sufficient ion conduction for maintaining reasonable power rates. To put this into perspective, such a high ionic conductivity is ∼50–100 times of that of an organic electrolyte used in existing lithium ion batteries. Our experiments and numerical analyses demonstrate how this configuration maintains performance.

## Results

### Proof of concept of Zn backside plating with half-cell

**Figure 2:**
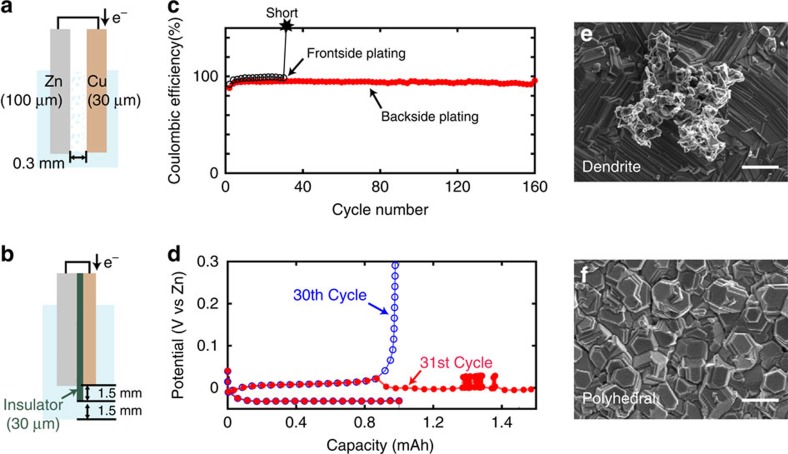
Electrochemical data of half-cells. Schematics of the half-cells with (**a**) conventional frontside- and (**b**) backside-plating cell configurations. (**c**) Corresponding high rate (20 mA cm^−2^) galvanostatic plating/stripping stability curves of the front and backside cells. (**d**) Plating/stripping curves of the conventional front-side cell at 30th and 31st cycle. (**e**) *Ex-situ* SEM image of Cu substrate surface at the 20th cycle of frontside and (**f**) backside-plating cell. Scale bars, 2 μm (**e**,**f**).

To prove that our concept of backside-plating configuration can avoid shorting, we made simple half-cells for both front (Fig. 2a) and backside-plating configurations (Fig. 2b) and performed electrochemical plating–stripping cycles. We used a Zn foil (100 μm in thickness) as reference and counter electrodes, and a Cu foil (30 μm) as a current collector for a working electrode. The electrodes are both round (0.8 cm in radius) and half-immersed into electrolyte in a round, airtight polycarbonate bottle (flooded cell) after cell assembly. The conductive electrode area in contact with the electrolyte is ∼1 cm^2^ (given by (0.8 cm)^2^ × π/2) and the upper half of the electrodes not in contact with electrolyte is connected to stainless steel current collectors. We use Cu foil as a current collector instead of Zn foil so that we can plate a known capacity of Zn onto Cu foil and then strip it away, allowing us to calculate meaningful Coulombic efficiency numbers. Cu also has a relatively high overpotential for the hydrogen evolution reaction (HER) and thus is used as current collector for Zn battery^13^,^16^. For a conventional frontside half-cell, a 0.3-mm gap between electrodes was allowed. For a backside-plating configuration half-cell, a film of insulator made of polypropylene (PP) (30 μm) is glued to Cu foil, to protect the frontside of Cu facing the cathode. To protect the edge of Cu substrate where current concentration takes place as indicated by our simulation (see Supplementary Fig. 1), an amorphous insulating carbon layer was sputtered around the edge^18^. The edge of the PP insulator extends out from the edge of Cu substrate by an additional 1.5 mm and thus immersed electrode area including the outside insulating ring is ∼1.4 cm^2^ (given by (0.8 cm+0.15 cm)^2^ × π/2). We used 6 M KOH aqueous solution saturated with ZnO as an electrolyte and all electrochemical measurements were done in airtight flooded cells.

In strong alkaline solutions such as 6 M KOH, Zn(OH)_2_ is reduced to Zn metal during plating as shown in equation (1) (ref. 19). Figure 2c,d show the Coulombic efficiencies of Zn plating (charge) and stripping (discharge) versus cycle number in half-cells for frontside- and backside-plating cells, respectively. For charging, Zn was plated with constant current density of 20 mA cm^−2^ until the total charge reached 1 mAh cm^−2^. Next, for discharging, Zn was stripped with the same constant current density until the voltage reached the cut-off voltage, 0.8 V versus Zn reference electrode for every cycle.

**Figure 3:**
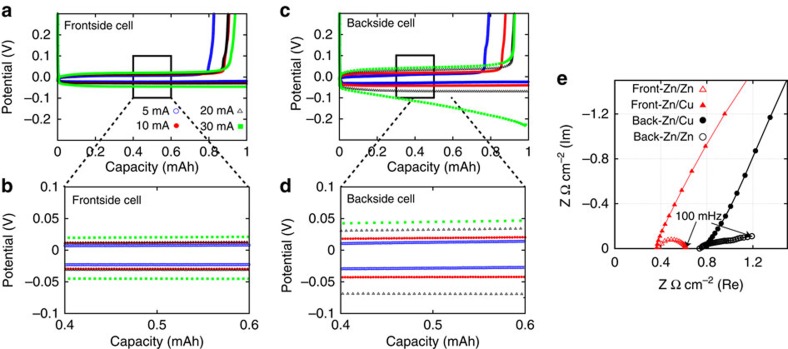
Comparison of power capability. Plating/stripping curves of (**a**,**b**) the frontside and (**c**,**d**) the backside-plating half-cells in different galvanostatic currents. 5, 10, 20 and 30 mA cm^−2^ were applied for both plating and stripping. (**e**) Corresponding impedance curves for the front and backside plating cells measured at open circuit potentials (∼0.9 V versus Zn). Half-cells use Zn and Cu as electrodes are denoted as Zn/Cu. In the same way, a cell using Zn for both electrodes are denoted as Zn/Zn. Frequency range for measurement is 1–100 MHz.

For the conventional frontside-plating configuration, a Coulombic efficiency of ∼96% was obtained until the 30th cycle, but a short occurred at the 31st cycle during Zn stripping due to dendrite formation on Zn counter electrode where Zn plating occurs. Corresponding charge–discharge voltage curves of the 30th and 31st cycle are shown in Fig. 2c. In the 30th cycle, the plating potential of Zn was at −30 mV versus Zn reference and the stripping was at 12 mV (*cf.* Fig. 3a,b). In the following Zn stripping at the 31st cycle, the curve showed an abrupt overpotential drop at ∼1 mAh and apparent capacity exceeding plating capacity with voltage noise around 1.3 mAh due to a short. Figure 2e shows the scanning electron microscopy (SEM) image of a dendrite formed on Cu surface after the Zn plating at the 20th cycle. Most of the Zn-plated surfaces were smooth with polyhedral type of Zn but formation of dendrites ranging from 5 to 10 μm in size was also confirmed (see Supplementary Fig. 2)^20^. The small dendrites would not penetrate immediately after the formation but they grow larger in size and cause a short. This is exactly the issue that Zn-based batteries face, hindering stable operation over hundreds of charge–discharge cycles.

In contrast, for backside plating, 160 cycles of stable plating stripping with Coulombic efficiency of 92% was achieved and no electrical shorts were observed. The plating potential of Zn was at −70 mV versus Zn reference and the stripping was at 50 mV (Fig. 3), which suggests that backside plating does not cause significant overpotential. Shown in Fig. 2f is the SEM image of the Cu surface after Zn plating at 20 cycles with backside-plating configuration. Only polyhedral Zn deposits were seen throughout the back surface of Cu. Polyhedra near the edge were larger than the ones at the terrace but there was no dendrite formation (see Supplementary Fig. 2). It should be emphasized that even if dendrites form in the back plating configuration, they do not result in an electrical short.

The reason for the slightly lower Coulombic efficiency in the backside-plating configuration compared with conventional one is HER caused by the carbon coating at the edge, which has a lower overpotential for HER than Cu. We confirmed that when the edge is not covered by carbon in backside-plating configuration, Coulombic efficiency becomes ∼96% between the 1st cycle and the 30th cycle, which is similar to the data obtained for frontside cells (see Supplementary Fig. 1e). To improve the Coulombic efficiency, alternative materials need to be explored in the future.

### Power capability of frontside- and backside-plating half-cells

Owing to the increased ionic pathway in the backside-plating configuration, it is essential to compare its power capability with that of the frontside. We performed galvanostatic electrochemical Zn plating and stripping at various current densities and measured the overpotentials as the key parameter for comparison. Figure 3a–d show the plating/stripping curves of 1 mAh cm^−2^ of Zn for conventional frontside- and backside-plating configuration cells, respectively, performed by half-cells at various current densities (5, 10, 20 and 30 mA cm^−2^).

For plating, overpotential for the frontside cell was only 30 mV at 20 mA cm^−2^. Backside-plating cells show slightly higher plating overpotential than frontside one and it was 70 mV at 20 mA cm^−2^. For stripping, the overpotentials for frontside and backside cells were ∼12 and 50 mV, respectively, at 20 mA cm^−2^ and there were no considerable difference between them. Larger overpotentials of the plating than of the stripping for both front and backside cells are presumably due to the activation energy required for Zn nucleation on Cu surface. When the current density is increased to 30 mA cm^−2^, we found that the backside cell shows relatively high overpotential over 200 mV, which may not be desired for practical use.

Thus, we found that backside-plating configuration allows a reasonably high current density of 20 mA cm^−2^ for plating without causing appreciable overpotential. The low overpotential at such a high current density of 20 mA cm^−2^ for backside-plating cell can be explained by the low solution resistivity (that is, the high ionic conductivity) of 6 M KOH aqueous electrolyte. The solution resistances of frontside and backside-plating cells were ∼0.4 and 0.8 Ω cm^−2^, respectively, as seen in Fig. 3e of impedance curves. The ionic conductivity of 6 M KOH zincate saturated aqueous solution was estimated to be ∼0.1 S cm^−1^ in our experiments, which is of the same order as reported values^17^.

### Ni-Zn full cell performance with backside-plating configuration

With the successful demonstration of backside Zn plating/stripping without dendrite formation or large overpotential at reasonably high current density, here we demonstrate a Ni-Zn full-cell with high power and long-cycle life as an example of the application of the backside-plating configuration concept. Electrochemical reactions involved here are given as follows:


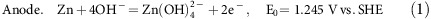



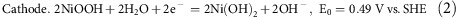



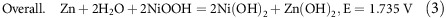


**Figure 4:**
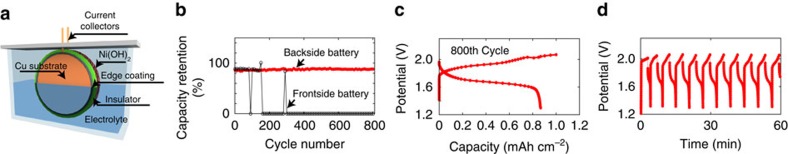
Ni-Zn full cells with backside-plating configuration. (**a**) Schematic representation of the backside-plating configuration full-cell battery. (**b**) Comparison of cycle stability curves of the Ni-Zn battery with the conventional frontside and backside battery cells. (**c**) Galvanostatic charge–discharge curves of Ni-Zn with the backside configuration (**c**,**d**). A steady charge–discharge cycle is achieved in the backside-plating configuration with high current density.

During charging, Ni(OH)_2_ at the cathode releases protons and is electrochemically converted to NiOOH. At the same time, zincate ions Zn(OH)_4_^2−^ are reduced and plated on the Cu substrate at anode. Discharge will progress in the opposite manner. The full-cell configuration is similar to the half-cell other than changing the cathode to β-Ni(OH)_2_ as illustrated in Fig. 4a. We confirmed the phase of β-Ni(OH)_2_ by X-ray diffraction (XRD) (see Supplementary Fig. 3). We used β-Ni(OH)_2_, as it has superior stability in strong alkaline solutions compared with α-Ni(OH)_2_. The theoretical capacity of β-Ni(OH)_2_ is 289 mAh g^−1^ and this material was successfully used as a cathode material in Ni-Cd batteries because of its high reliability. It is still used in a Ni-MH battery for hybrid vehicles. When combined with a Zn electrode, this battery offers one of the highest overall potentials (∼1.7 V) in aqueous batteries. The specific energy (calculated based on active material weight only) can reach ∼372 Wh kg^−1^.

Figure 4b shows the cycle stability curves of Ni-Zn full cells with frontside- and backside-plating configurations, respectively. The battery is charged up to total charge capacity of 1 mAh cm^−2^ and discharged to cutoff voltage of 1.3 V with current density of 20 mA cm^−2^ (20 C or 3 min for charge and discharge, respectively) for every cycle. Capacity retention, which is equivalent to Coulombic efficiency, is calculated by the following equation:

Capacity retention (%)=discharge capacity/charge capacity (fixed to be 1 mAh cm^−2^) × 100 (4)

The frontside cell shows a spike in stability curve at around 100 cycles due to a short and cycled until about 150 cycles when it shorts again. Around the 280th cycle, there is a short recovery of the capacity, presumably due to the detachment of the zinc dendrite that was bridging the two electrodes, and then the cell shorts again at later cycles.

In contrast, the backside cell shows expected highly stable cycling more than 800 times free of any indication of shorting. 88% capacity retention was maintained at 800 cycles, which has never been achieved before with conventional frontside batteries, except using a flow cell system with the electrode separation much larger than the static cells^21^,^22^. Galvanostatic charge–discharge curves of Ni-Zn with backside-plating configuration at the 800th cycle are shown in Fig. 4c,d. The average charge and discharge voltage were found to be 1.95 and 1.65 V, respectively, which are typical for Ni-Zn full-cell battery. Thus, the specific energy density for the full cell shown in Fig. 4 was estimated to be ∼246 Wh kg^−1^ based on the active materials' weight (∼4.8 mg of commercial Ni(OH)_2_ and ∼1.2 mg of Zn) for both conventional and backside-plating configuration Ni-Zn full-cell batteries tested (1 mAh × 0.9 × 1.65 V × (4.8 mg+1.2 mg)^−1^). In general, a battery suffers a decrease of capacity in real devices due to the volume of inactive components such as separator, current collectors, packaging and so on, and volumes of these parts need to be small for achieving the energy density closer to the theoretical one. In the backside-plating configuration, it is also important to reduce inactive volume for the same reason. In the following section, we discuss the energy density considering not only the active materials' weight but also the volume of electrolyte over the backside of Cu foil required for ion transport in the backside-plating configuration.

## Discussion

Stable operation for more than 800 cycles (as demonstrated in the Ni-Zn full-cell batteries) was achieved with the backside-plating configuration. Here we discuss the importance of insulating the Cu foil edge in avoiding dendrite formation there, as well as the importance of solution thickness above the Cu surface for avoiding dendrite formation near the centre of backside of Cu surface. In the backside cell, we covered the edge of the Cu substrate by a carbon insulating layer. The key role of this layer, which is amorphous and highly resistive^18^, is to avoid preferential plating of Zn on the very edge. Metallic Cu has higher electrical conductivity than amorphous carbon and therefore Zn will be plated onto Cu substrate rather than the protected edge. Our numerical analysis results indicated a certain current concentration at the edge (see Supplementary Fig. 1a–d) and we confirmed the dendrite formation at the edge of Cu after repeated cycles of Zn plating and stripping when the edge of Cu was not protected (see Supplementary Fig. 4). Accordingly, the cycle stability curve of the half-cell without carbon coating shows small spikes and the curve became noisy (see Supplementary Fig. 1e). Although suggesting an intermittent and less disruptive process in comparison with the clear signs of shorting in the conventional cells, this unstable cycling behaviour nonetheless needs to be avoided from a practical viewpoint.

In backside-plating configuration, certain thickness of electrolyte above the Cu is necessary to supply enough ions for the whole back surface, in particular the surface of the centre far from the edge. If the thickness of the solution is not adequate for the distance from the edge to the centre of the Cu back surface, ions would be reduced at around the edge side during charging and most probably a thicker layer of Zn would be formed, resulting in depletion of ions near the centre of the surface. Such depletion of ions can also cause the formation of dendrites as reported^11^,^23^,^24^. Hence, the optimal design should allow a certain solution thickness to avoid such depletion of ions to support sufficient ion supply, yet it should not be too thick, to maintain useful energy density of the cell.

To investigate how thick the electrolyte must be for plating only smooth Zn for the whole back surface, we prepared the backside-plating half-cells with different solution thicknesses ranging from 0.5 to 2.0 mm by placing polycarbonate film above the solution and plated 1 mAh cm^−2^ of Zn with fixed current density of 20 mA cm^−2^. We found that a majority of Zn deposits were polyhedral/boulder structures within ∼2 mm from the edge of Cu for the cell with 0.5 mm of solution thickness. Beyond 2 mm from the edge to the Cu back surface centre, Zn dendrites were formed due to the depletion of Zn ions (see Supplementary Fig. 5a). We gradually increased the solution thickness so that the whole back surface can be covered by Zn polyhedral/boulder structure and found that 2 mm of solution thickness was required for plating polyhedral/boulder Zn at the centre of backside of Cu surface (see Supplementary Fig. 5b).

Next, we prepared the half-cells with a smaller solution thickness of 50 μm, which is secured by inserting the PP battery separator placed between the back surface of Cu and polycarbonate outer film. We plated Zn corresponding to the capacity of 1 mAh cm^−2^ with the different current densities as shown in Supplementary Fig. 6a (2, 5 and 10 mA cm^−2^). At the current density of 10 mA cm^−2^, the overpotential was ∼0.15 V at the end of plating. When the current density was decreased to 2 mA cm^−2^, the overpotential was ∼0.05 V and the whole back Cu surface was plated with polyhedral/boulder Zinc (Supplementary Fig. 7). With this solution thickness and plating capacity, the energy density was calculated to be higher than 150 Wh kg^−1^ (Supplementary Fig. 6b); higher values are possible if a fraction of active materials is increased, which may be possible through engineering efforts.

When the radius of Cu foil was 0.8 cm, the solution thickness was 2 mm and the current density was 20 mA cm^−2^; the whole back surface was plated with polyhedral/boulder Zn with no dendrites (Supplementary Fig. 5). Thus, the ratio of the radius of the Cu foil to the solution thickness for plating polyhedral/boulder Zn without a dendrite can be calculated to be 4:1. In the same way, at the current density of 2 mA cm^−2^, the ratio is calculated to be 160 (0.8 cm) to 1 (50 μm) based on the SEM observations shown in Supplementary Fig. 7. The ratio of the radius of the Cu foil to the solution thickness for preventing dendrite formation would be helpful for designing backside-plating configuration batteries. Finally, we note that the geometric advantage of backside-plating configuration prevents shorting problem even if dendrite formation takes place during Zn plating. Thus, it may not be always necessarily required to plate only polyhedral Zn throughout the surface in practical use. Therefore, the solution thickness might be reduced further for better energy density.

In summary, we developed a backside-plating configuration for avoiding a short of Zn-based batteries. As a proof-of-concept, we demonstrated highly stable 800 charge–discharge cycles with a Ni-Zn full-cell battery under high rate (20 mA cm^−2^) using 6 M KOH zincate aqueous solution in backside-plating configuration. We found that the backside-plating configuration requires a certain thickness of solution above the plating substrate for homogenous plating of Zn throughout the substrate. We prepared half-cells with different solution thickness (2 mm and 50 μm) and applied different current densities (20 and 2 mA cm^−2^) to estimate the ratio of the radius of the Cu foil to the solution thickness, as an attempt to establish the design rule for the backside-plating configuration batteries. Many different approaches have been studied to avoid shorting due to dendrite formation but further improvements are needed. The backside-plating method can provide a way for turning high-capacity electrodes limited to primary batteries by dendrite formation into reliable components of rechargeable systems.

## Methods

### Material characterization by uses of *ex situ* SEM and XRD

SEM images were taken using a FEI XL30 Sirion SEM (accelerating voltage 5 kV). XRD patterns were recorded on a PANalytical X 'Pert Diffractometer operated at 45 kV and 40 mA at the wavelength of Cu Kα radiation (*λ*=0.15406, nm). After the plating of Zn, substrates are detached from the insulator and outer package by ethanol, thoroughly washed by deionized water (18 M Ω) and immediately dried by blowing air. Dried substrate was glued to slide glass for XRD measurements.

### Electrochemistry

Galvanostatic experiments were performed using multi-channel potentiostat, VMP3 (BioLogic). For half-cell galvanostatic experiments, 1 mAh of Zn (corresponding to ∼1.2 mg of Zn) was plated/charged onto Cu substrate, followed by stripping of Zn up to 0.8 V versus Zn reference electrode for every cycle. For full cells, 1 mAh is charged and then discharged to 1.3 V of battery potential for every cycle. For both experiments, electrode surface touching the electrolyte is designed to be 1 cm^2^.

KOH (6 M) zincate saturated aqueous solution was prepared by mixing 500 ml of deionized water, 168 g of KOH and 20.35 g of ZnO (Sigma-Aldrich), and stirred for 12 h at 70 °C. The mixed solution was bubbled by N_2_ gas for 2 h and centrifuged before use. Zn (Alfa Aesar) and/or Mercury Oxide Electrode (Koslow 5088) were used as reference electrodes. Airtight beaker cells with 30 ml of electrolyte were used for measurements. Cathode, anode and reference electrodes are sunk into the electrolyte during electrochemical measurements.

### Full-cell preparation

Cathodes are prepared as follows: 700 mg of Ni(OH)_2_ (Sigma-Aldrich), 200 mg of Acetylene black, 100 mg of polyvinylidene difuluoride (Kynar HSV900) and 10 ml of *N*-methyl-2-pyrrolidone (Sigma-Aldrich) were mixed and stirred for 12 h at room temperature. The obtained slurry was dip coated on current collector of Ni foam (2 mm thick) and dried at vacuum oven 80 °C. Dried Ni foam was pressed in to 0.1 mm to be used as cathode. Approximately 4.8 mg commercial Ni(OH)_2_ is loaded on the prepared cathode. Carbon coater (EMS150R ES) was used for sputtering the amorphous carbon nano layer. Carbon fibres were used as the evaporation target. The evaporation chamber was pumped down to 5 × 10^−2^ mbar before an out gassing current of 30 A was passed through the carbon fibres. After out-gas recovery, a pulse current was passed through the fibre to allow flash evaporation of carbon. The pulse current was set to 60 A for 20 s, with a 10-s interval between pulses. All the procedure is the same as that in ref. 18. A mask was used to coat the carbon layer on only the edge of Cu substrate. The edge-protected Cu substrate was then glued to PP film by Polyvinyl butyral (Santa Cruz Biotechnology).

### Numerical analysis

COMSOL multiphysics (COMSOL Inc.) was used, to estimate current density profile in the electrolyte and on the surface of the copper electrode. The two-dimensional axisymmetric structure of simulated electrolyte consists of the surface of zinc electrode as an anode, insulating surface and the surface of copper electrode a cathode. The radius of the copper electrode is 8 mm, the thickness of zinc electrode is 1 mm and the height of electrolyte above the copper surface for zinc deposition is varied from 50 μm to 2 mm. The input current density on zinc electrode is varied from 1 to 20 mA cm^−2^. For the estimation of current density profile of zinc ion on the copper electrode, Nernst–Planck equation is employed as a governing equation in COMSOL Multiphysics. This mode of simulation counts diffusion and migration due to the electric field as a regime of the transportation of zinc ion during electrochemical deposition. The given diffusivity of zinc ion in the aqueous electrolyte is 6 × 10^−6^ cm^2^ s^−1^. The simulation results visualize the colour map of current density, contour lines of overpotential and the streamlines (red) of zinc ion flow.

### Data availability

The authors declare that the data supporting the findings of this study are available within the article and its Supplementary Information File.

## Additional information

**How to cite this article:** Higashi, S. *et al*. Avoiding short circuits from zinc metal dendrites in anode by backside-plating configuration. *Nat. Commun.* 7:11801 doi: 10.1038/ncomms11801 (2016).

## Supplementary Material

Supplementary Information Supplementary Figures 1 - 7

## Figures and Tables

**Figure 1:**
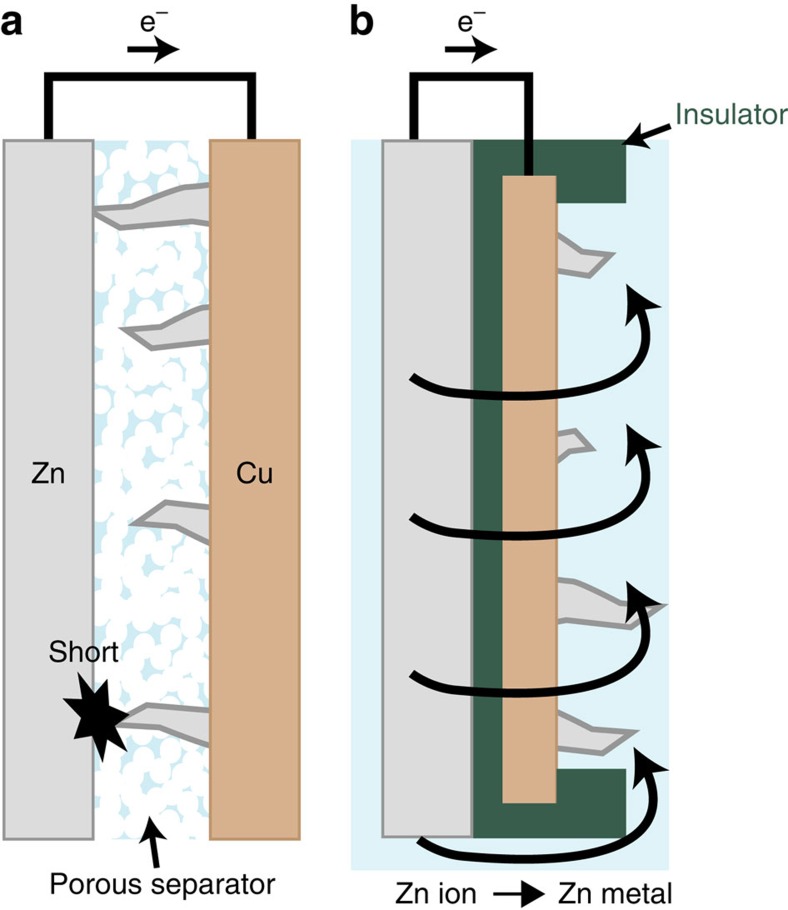
Scheme of backside-plating configuration for avoiding internal shorts. Schematic representation of (**a**) conventional frontside- and (**b**) backside-plating configuration cells. In conventional configuration, electrodes face each other separated by a porous polymer separator, which causes a battery short due to the formation of dendrites under repeated cycles. In the backside-plating configuration, an insulator protects the front side and edge of Cu, allowing Zn to grow on the backside of substrate Cu. Backside cell is configured to have aqueous electrolyte between Zn and Cu back surface.
