# Supplementary material for: Avoiding short circuits from zinc metal dendrites in anode by backside-plating configuration
Source: Nat Commun. 2016 Jun 6;7:11801. doi: 10.1038/ncomms11801 (PMC4897743; doi:10.1038/ncomms11801)
Supplement: Supplementary Information — Supplementary Figures 1 - 7 [file ncomms11801-s1.pdf]

Supplementary Figures

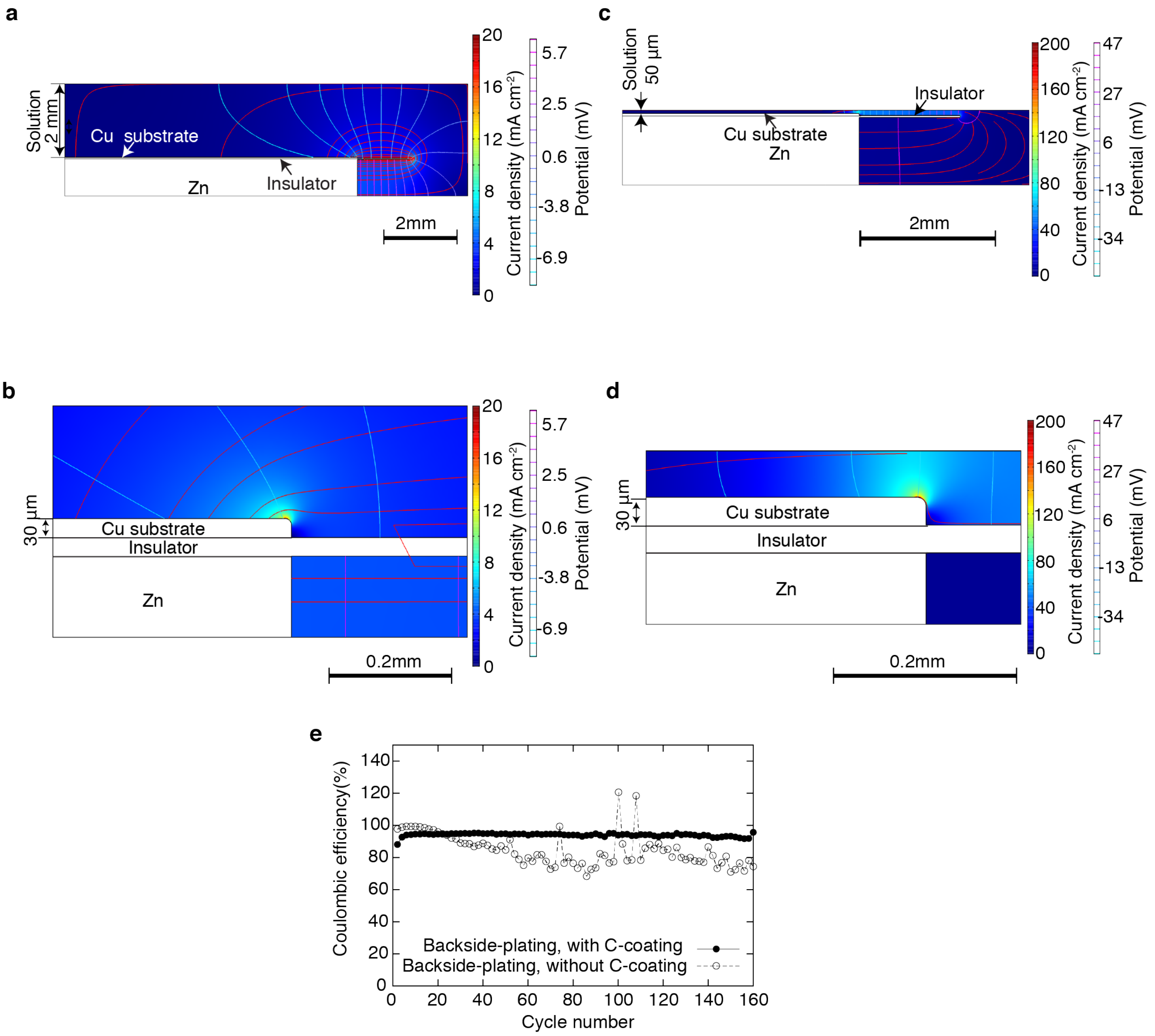

Supplementary Fig. 1. Current density profiles for backside-plating configuration cells and the cycle stability curve with and without carbon coating.

Current density profiles of Zn ions on the copper electrode at an input current of 4 mA cm^{-2} for solution thickness of (**a, b**) 2 mm and (**c, d**) 50 µm. **b** and **d** are magnified view of **a** and **c**, respectively. (**e**) Plating and stripping stability curves of edge protected and non-protected backside-plating half-cells with a solution thickness of 2 mm. Current density for plating and stripping cycles is 20 mA cm^{-2} and 1 mAh cm^{-2} of Zn is plated followed by stripping up to 0.8 V versus Zn reference electrode for every cycle.

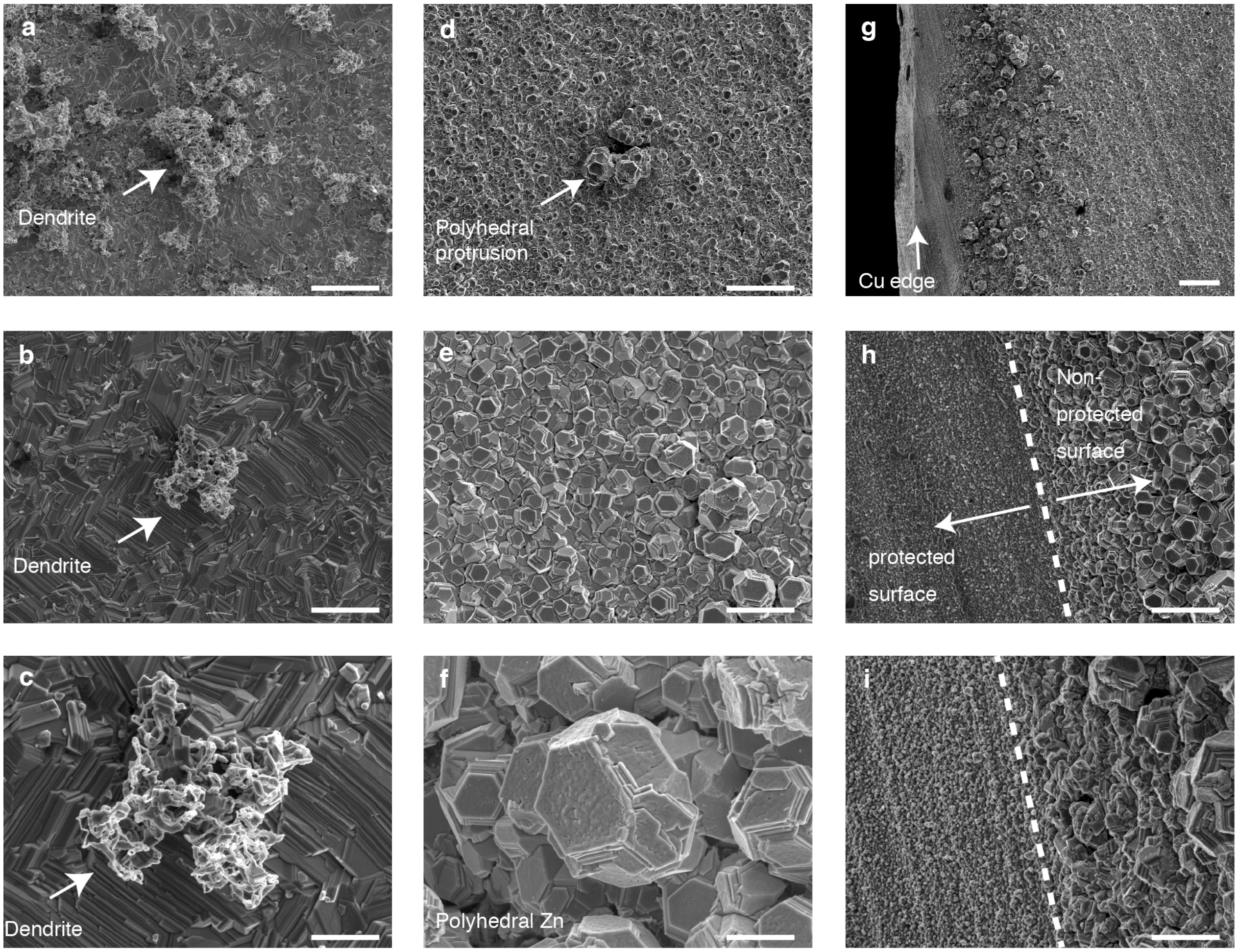

Supplementary Fig. 2| *Ex-situ* SEM images of substrate Cu after Zn plating.

Ex-situ SEM images of Cu substrates for front-side (**a-c**) and backside-plating cells (**d-f**) at different magnifications. Dendrites are randomly formed at the surface in the front-side cell. Dendrite formation was not confirmed in the backside-plating cell. Closer look of edge of Cu revealed relatively large polyhedral Zn deposits were formed at about 20 μm from the edge of Cu in the backside-plating configuration cells (**g-i**). Scale bars in (**a, d**) and (**g**) are 10 μm and 20 μm, respectively, and (**b, e, f**) and (**c, f, i**) are 5 and 2 μm, respectively.

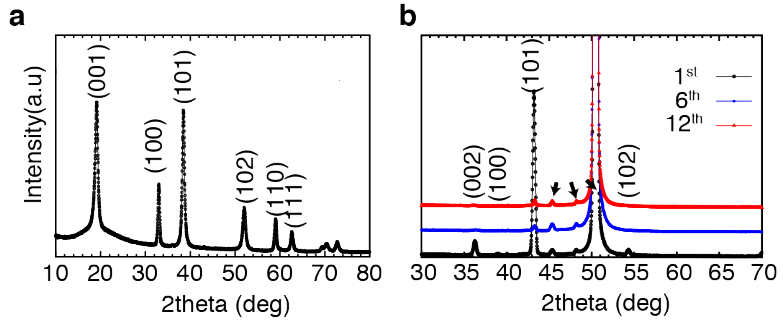

Supplementary Fig. 3| *Ex-situ* XRD patterns of commercial β-Ni(OH)$_2$ and Cu substrate at different cycle number.

(**a**) *Ex-situ* XRD pattern of β-Ni(OH)$_2$. (**b**) *Ex-situ* XRD patterns of the Zn plated Cu substrate obtained for Zn (1 mAh cm^{-2}) plated Cu substrate at 1st cycle (black), Zn stripped Cu substrate at 6th (blue) and 12th (red) cycle. Zn derived XRD signals were significantly weakened after stripping, indicating Zn is dissolved into electrolyte. XRD patterns of 6th and 12th cycle are almost identical, indicating accumulation of crystalline byproduct is not significant during the plating/stripping cycles. Arrows indicate Cu substrate peaks.

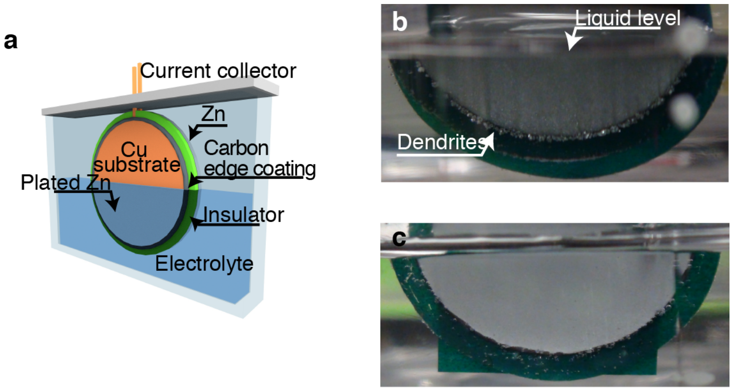

Supplementary Fig. 4| Schematic and optical images of backside-plating half-cells.

(**a**)Schematic representation of half-cell with backside-plating configuration. Backside-plating half-cell after plating 1 mAh of Zn at 150th cycles for (**b**) non-protected and (**c**) protected.

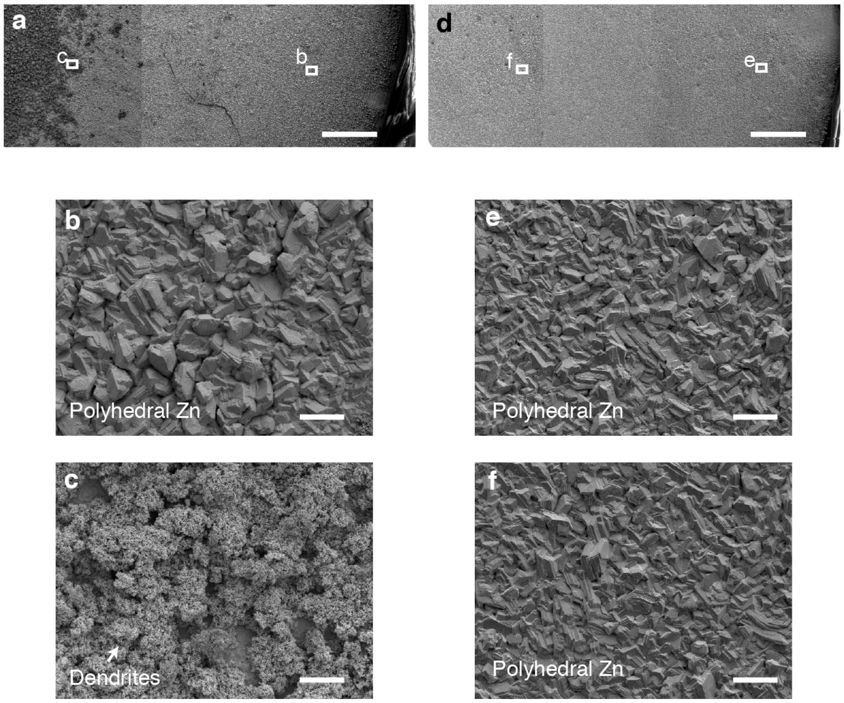

Supplementary Fig. 5| Ex-situ SEM observations and comparison of morphology of plated Zn at different solution thickness above the Cu back surface.

(**a-c**) 5 mm and (**d-f**) 2 mm solution thickness. (**b**) Magnified SEM image at point b of **a**. (**c**) Magnified image at point c of **a**. (**e**) Magnified SEM image at point e of **d**. (**f**) Magnified SEM image at point f of **d**. Scale bars are 500 and 20 μm in **a, d** and **b, c, e, f** respectively.

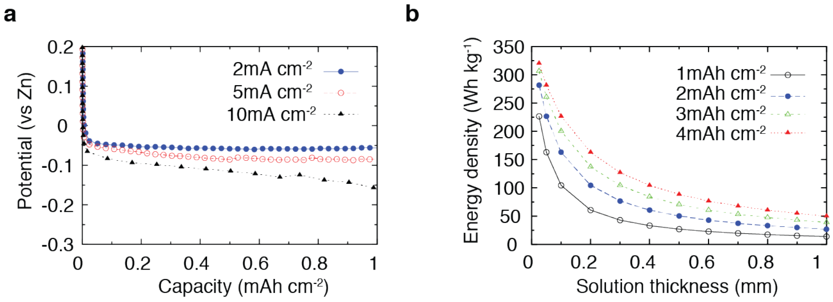

Supplementary Fig. 6 | Plating potential profiles for the half-cells with a solution thickness of 50 µm and energy density dependence of Ni-Zn full cell.

(**a**) Potential profiles of the backside-plating configuration half-cells at different current densities (2, 5 and 10 mA cm^{-2}) during Zn plating. A 50 µm thick separator is placed between the Cu back surface and polycarbonate film. (**b**) Calculated specific energy density of Ni-Zn battery based on the mass of both active materials and electrolyte for different solution thicknesses of electrolyte.

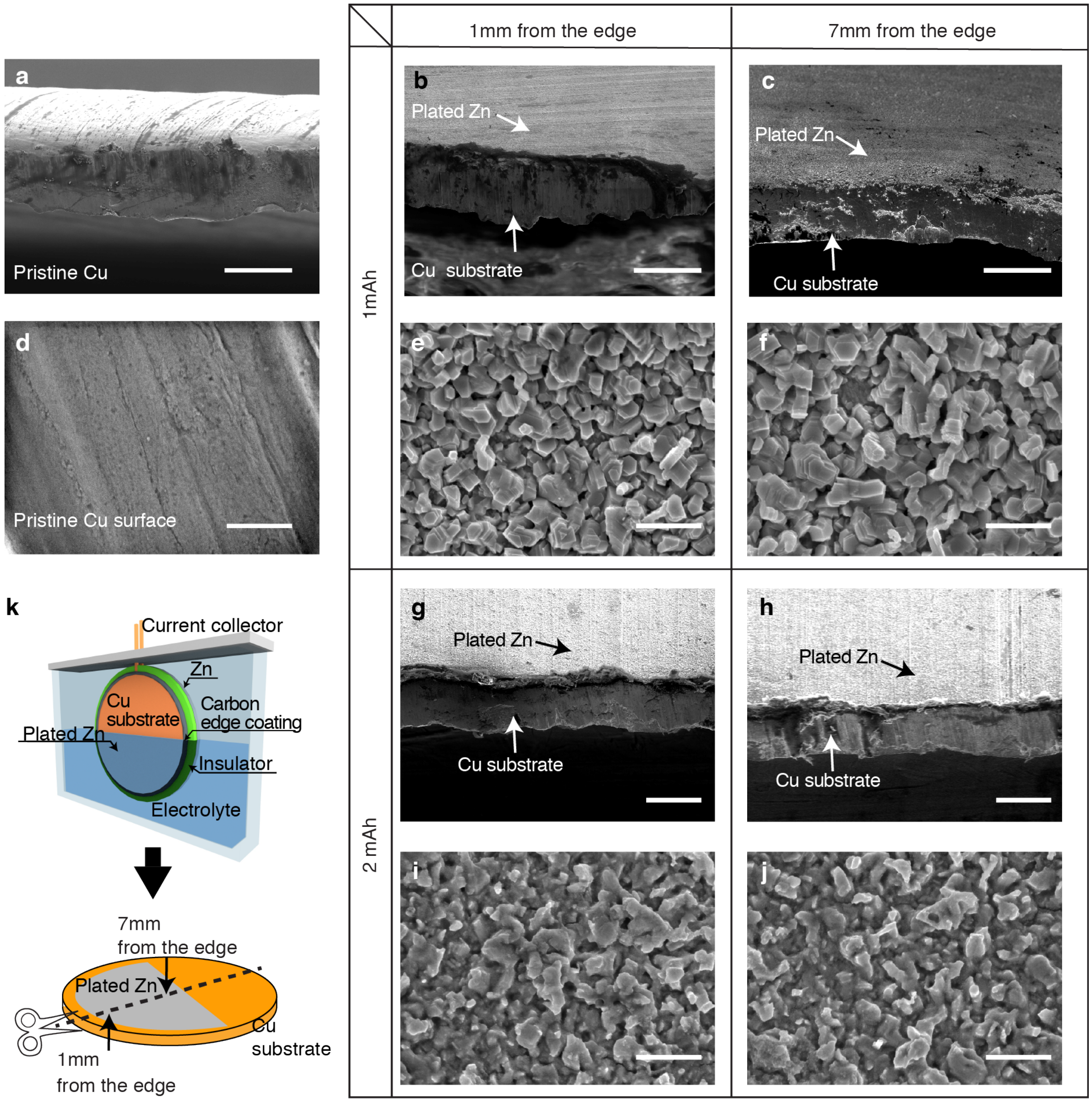

Supplementary Fig. 7 | Cross sectional and top view SEM images of Zn plated Cu substrate with backside-plating configuration.
(**a**, **b**, **c**, **g**, **h**) Cross sectional and (**d**, **e**, **f**, **i**, **j**) top view SEM images of Zn plated Cu substrate. (**a, d**) Pristine Cu. (**b**, **c**, **e**, **f**) 1 mA cm^{-2} of Zn plated Cu and (**g**, **h**, **i**, **j**) 2 mA cm^{-2} of Zn plated Cu. Current density of 2 mA cm^{-2} was applied for the half-cells with the solution thickness of 50 μm (see supplementary Fig. 6b for the potential profiles at the current density of 2 mA cm^{-2}). Cross sectional SEM images were taken using a tilted SEM sample holder with an angle of 45 degree (Thus the scale bar in vertical direction is $1/\sqrt{2}$ of the horizontal one). SEM images of **b**, **e**, **g**, **i** and **c**, **f**, **h**, **j** were taken at 1 and 7 mm from the edge, respectively. Scale bars in **a**, **b**, **c**, **g**, **h** and **d**, **e**, **f**, **i**, **j** are 20 μm and 500 nm, respectively. (**k**) Schematics of the experimental setup for the half-cell and sample preparation for cross sectional SEM.
